# Rabbit M1 and M2 macrophages can be induced by human recombinant GM‐CSF and M‐CSF

**DOI:** 10.1002/2211-5463.12101

**Published:** 2016-08-15

**Authors:** Kazuyoshi Yamane, Kai‐Poon Leung

**Affiliations:** ^1^ US Army Dental and Craniofacial Trauma Research and Tissue RegenerationInstitute of Surgical ResearchFort Sam HoustonTXUSA; ^2^ Department of BacteriologyOsaka Dental UniversityHirakata‐shiOsakaJapan

**Keywords:** M1/M2, macrophages, monocytes, *Oryctolagus cuniculus*, polarization, rabbit

## Abstract

Macrophages can change their phenotype in response to environmental cues. Polarized macrophages are broadly classified into two groups: classical activated M1 and alternative activated M2. Characterization of human macrophages has been widely studied, but polarized macrophages in rabbits have not been characterized. We characterized rabbit macrophages that were polarized using human recombinant GM‐CSF and M‐CSF. GM‐CSF‐treated macrophages had higher mRNA expression of proinflammatory cytokines (M1 phenotype) than did the M‐CSF‐treated counterpart. By contrast, high levels of TGF‐β and IL‐10 expression (M2 phenotype) were found in M‐CSF‐treated macrophages. The present study may be useful to understand roles of polarized macrophages in rabbit disease models.

**Abbreviations**
ACTB: beta‐actin
FBS: fetal bovine serum
GAPDH: glyceraldehyde 3‐phosphate dehydrogenase
GM‐CSF: granulocyte macrophage colony‐stimulating factor
G‐MΦs: GM‐CSF‐treated macrophages
IL: interleukin
iNOS: inducible nitric oxide synthase
LPS: lipopolysaccharide
M‐CSF: macrophage colony‐stimulating factor
MHC: major histocompatibility complex
M‐MΦs: M‐CSF‐treated macrophages
mRNA: messenger RNA
NO: nitric oxide
NOS: nitric oxide synthase
PBMCs: peripheral blood mononuclear cells
PBS: phosphate‐buffered saline
TGF: transforming growth factor
Th: T helper
TNF: tumor necrosis factor

Rabbits (the family of Leporidae) play important roles both in agriculture and as medical research animals. They are widely used for basic and clinical research of human diseases because they are phylogenetically more closely related to primates than are rodents. In addition, rabbits share many advantages with rodents such as their small size, shorter reproduction times, ease of breeding, and ease of maintaining colonies due to large litter sizes 1. Among various strains, the New Zealand white rabbit (*Oryctolagus cuniculus*) is commonly being used for research of diseases such as cancer 2, atherosclerosis 3, Alzheimer's disease 4, and eye research 5. Moreover, owing to similarities of rabbit ear skin to that of human skin, New Zealand white rabbit ear models have been established for wound healing research. Rabbits are also used to determine inflammatory responses of wounds to bacterial biofilm infection 6, 7, 8 and diabetic neuroischemic wound healing 9.

Inflammation involves the interplay between inflammatory mediators, neutrophils, and immune cells including macrophages and dendritic cells. Macrophages play a crucial role in inflammation in response to microorganisms and injuries. Moreover, they have homeostatic and metabolic functions including iron‐control, and tissue repair and remodeling 10, 11. Macrophages display remarkable plasticity and can change their physiology in response to environmental cues 12. Their phenotypical changes are called polarization. Polarized macrophages are broadly classified into two groups, classical activated M1 and alternative activated M2 macrophages. M1 macrophages are characterized by a proinflammatory phenotype, promotion of T helper (Th)1 immune response while M2 display promotion of the Th2 immune response 13. M2 macrophages play different roles in various phases of tissue remodeling and repair, suppression of inflammation, and elimination of parasites 11. *In vitro* studies using cultured macrophages have characterized polarization profiles in mice and humans, and have shown them to be common in phenotypes but different in gene regulation for polarization 14. However, at present, little is known about the polarization of macrophages in other model animals such as rabbits. Extensive M1/M2 characterization of macrophages in multiple model animals would greatly supplement existing knowledge and aid in determining mechanisms of diseases. Understanding the role of rabbit M1 and M2 macrophages in tissues is important to develop advanced procedures for diagnosis and treatment as well as new experimental models in rabbit.

In this report, we focused on *in vitro* rabbit macrophage polarization from peripheral blood mononuclear cells by human granulocyte macrophage colony‐stimulating factor (GM‐CSF) and human macrophage colony‐stimulating factor (M‐CSF; also known as colony‐stimulating factor 1). Our efforts involved determinations of cytokine gene expression, nitric oxide synthase (NOS) and arginase activity, phagocytic capacity, and cell surface receptor expression in rabbit polarized macrophages.

## Materials and methods

### Isolation of macrophages from peripheral blood

Fresh blood collected from female New Zealand white rabbits (Covance Research Products, Denver, PA, USA) was used in this study. Peripheral blood mononuclear cells (PBMCs) including monocytes were isolated using OptiPrep (Axis‐Shield, Oslo, Norway) according to a modified manufacturer's protocol for rabbit blood. Twofold diluted rabbit's blood was layered on OptiPrep density solution. After density gradient centrifugation at 700 ***g*** for 20 min with nonaccelerator and nonbrake modes, isolated PBMCs were washed two times with phosphate‐buffered saline (PBS; GE Healthcare Life Sciences Hyclone Laboratories, Logan, UT, USA) by centrifugation at 250 ***g*** for 10 min. Cells were resuspended in RPMI‐1640 medium (Life Technologies, Grand Island, NY, USA) with 10% fetal bovine serum (FBS; GE Healthcare Life Sciences Hyclone Laboratories) and 1% penicillin/streptomycin (Life Technologies) at a concentration of 2 × 10^6^ cells·mL^−1^. Subsequently, cell suspensions were transferred to T75 tissue culture flasks (Sarstedt, Newton, NC, USA), four‐well chamber slides (Thermo Fisher Scientific, Rochester, NY, USA), or 24‐well flat bottom tissue culture plates (Becton Dickinson, Flanklin Lakes, NJ, USA). After incubation for 2 h at 37 °C in humidified 5% CO_2_ in a gas incubator (Galaxy 170R; Eppendorf, Enfield, CT, USA), nonadherent cells were removed by washing with PBS 15.

### *In vitro* polarization of macrophages

**Figure 1:**
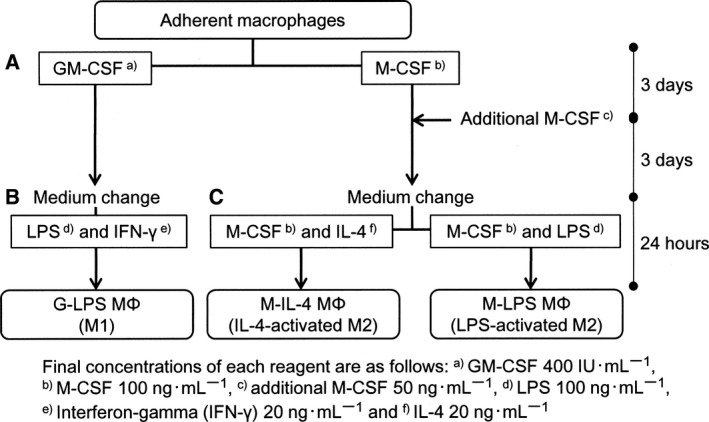
Schematic overview of the rabbit macrophage differentiation protocol. Adherent macrophages in RPMI‐1640 medium supplemented with 10% FBS were primed with GM‐CSF (G‐MΦ) or M‐CSF (M‐MΦ) (A). After changing medium to RPMI‐1640 supplemented with 5% FBS, G‐MΦs were stimulated with LPS and interferon‐gamma (B; G‐LPS‐MΦ). M‐MΦs were stimulated with M‐CSF and IL‐4 (M‐IL‐4‐MΦ) or with M‐CSF and LPS (M‐LPS‐MΦ) (C).

To generate *in vitro* polarization (Fig. 1), adherent macrophages were cultured for 6 days in RPMI‐1640 medium with 10% FBS and 1% penicillin/streptomycin supplemented with either: (a) recombinant human GM‐CSF (Peprotech, Rocky Hill, NJ, USA; 400 IU·mL^−1^) that induced polarized M1 macrophages (G‐MΦs); or (b) human M‐CSF (Peprotech; 100 ng·mL^−1^) that induced polarized M2 macrophages (M‐MΦs). All percentages and concentrations are referred to those present in the final incubation medium. The cells were incubated for 6 days at 37 °C in humidified 5% CO_2_ in a gas incubator without shaking. During this incubation period, 3 days after inoculation, an additional M‐CSF (50 ng·mL^−1^) was added to M‐MΦs without changing media. For stimulation of the cells after 6 days of incubation, G‐MΦs were exposed to a fresh RPMI‐1640 medium supplemented with FBS (5%), lipopolysaccharide (LPS) obtained from *Escherichia coli* serotype O55: B5 (Sigma‐Aldrich, St. Louis, MO, USA; 100 ng·mL^−1^), and recombinant rabbit interferon gamma (Kingfisher Biotech, St. Paul, MN, USA; 20 ng·mL^−1^) for 24 h (G‐LPS‐MΦs). Similarly, for the M‐MΦs, RPMI‐1640 medium supplemented with FBS (5%), M‐CSF (100 ng·mL^−1^), and recombinant rabbit interleukin (IL)‐4 (R&D Systems, Minneapolis, MN, USA; 20 ng·mL^−1^) (M‐IL‐4‐MΦs), or a combination of M‐CSF (100 ng·mL^−1^) and lipopolysaccharide (100 ng·mL^−1^; M‐LPS‐MΦs) were used for 24 h at 37 °C in humidified 5% CO_2_.

### RNA extraction and reverse transcription real‐time quantitative PCR (RT‐qPCR) analysis

Total RNA of polarized MΦs cells was extracted using a RNeasy Mini Kit (Qiagen, Valencia, CA, USA) based on the manufacturer's instructions. Contaminating genomic DNA was removed from RNA samples using Turbo DNase (Life Technologies). About 1 ng of isolated RNA was reverse‐transcribed to cDNA using an iScript cDNA Synthesis Kit (Bio‐Rad Laboratories, Hercules, CA, USA) in an iCycler Thermal Cycler (Bio‐Rad). Complementary DNA generated was mixed with 300 nm each of gene‐specific primers (Table 1) and 1× iQ SYBR Green Supermix (Bio‐Rad). The messenger RNA (mRNA) expression of various macrophage markers was assessed using stepone plus real‐time pcr system (Life Technologies). Data were analyzed with stepone Software v.2.0 (Life Technologies) via the comparative C_T_ method (ΔΔC_T_) using the house‐keeping genes, glyceraldehyde 3‐phosphate dehydrogenase (GAPDH) or beta‐actin (ACTB) gene, for normalization of mRNA expression. Gene expression of each sample was represented by a ratio of ΔC_T_ to that of M‐IL‐4‐MΦ.

**Table 1:** Primers used for real‐time PCR

Gene	Forward primer sequence	Reverse primer sequence	Reference
Beta‐actin	5′‐AGGAGAAGCTGTGCTACGTG	5′‐CAGGAAGGAGGGCTGGAACA	This study
GAPDH	5′‐AGGTCATCCACGACCACTTC	5′‐GTGAGTTTCCCGTTCAGCTC	8
IL‐1 beta	5′‐CCACAGTGGCAATGAAAATG	5′‐AGAAAGTTCTCAGGCCGTCA	8
IL‐6	5′‐GAACAGAAAGGAGGCACTGG	5′‐CTCCTGAACTTGGCCTGAAG	31
IL‐10	5′‐CAAGCCTTGTCGGAGATGAT	5′‐TTTTCACAGGGGAGAAATCG	This study
IL‐12 p35	5′‐AAGGCCAGACAAACTCTAGAATTC	5′‐TTGGTTAACTCCAGTGGTAAACAGG	1
IL‐12/IL‐23 p40	5′‐CTCCGAAGAAGATGGCATTACC	5′‐TCTCCTTTGTGGCAGGTGTATTG	1
TGF‐beta	5′‐CAGTGGAAAGACCCCACATCTC	5′‐GACGCAGGCAGCAATTATCC	1
TNF‐alpha	5′‐GTCTTCCTCTCTCACGCACC	5′‐TGGGCTAGAGGCTTGTCACT	8

GAPDH, glyceraldehyde 3‐phosphate dehydrogenase; IL, interleukin; iNOS, inducible nitric oxide synthase (nitric oxide synthase 2); TGF, transforming growth factor; TNF, tumor necrosis factor.

### NOS activity assay

Cells (2 × 10^6^) on 24‐well plates were washed with cold PBS and lyzed. NOS activities of differentiated cells were determined by measuring the accumulation of nitrate and nitrite, stable degradation products of nitric oxide (NO) metabolism. Total protein concentrations of cell lysates were measured using absorbance at 280 nm (NanoDrop 2000c; Thermo Scientific, Wilmington, DE, USA) and diluted with cold PBS to same protein level. After reduction of nitrate to nitrite, nitrite levels were determined using an Ultra Sensitive Assay for Nitric Oxide Synthase Kit (Oxford Biomedical Research, Oxford, MI, USA). Spectrophotometric readings were taken and recorded using a plate reader (SynergyHT; BioTek, Winooski, VT, USA) with the wavelength set at 540 nm. NOS activity was described as the amount of nitrite based on a standard curve of known sodium nitrite concentrations. Activities were indicated as the mean of five independent wells. Experiments were biologically repeated twice using blood from two rabbits. Gene expression patterns of inducible nitric oxide synthase (iNOS) in the differentiated cells were compared by RT‐qPCR as described above using specific primers (Table 1).

### Arginase activity assay

Cells (2 × 10^6^) on 24‐well plates were washed with cold PBS and lyzed. Arginase activity in cell lysates was measured using an Arginase Activity Colorimetric Assay Kit (BioVision, Milpitas, CA, USA) according to the manufacturer's instructions. By heating cells at 37 °C for 30 min, the concentration of intermediate generated from the conversion of L‐arginine was measured spectrophotometrically using a plate reader with the wavelength set at 570 nm. The activity was described as the amount of hydrogen peroxide generated by arginase. One unit of arginase is the amount of enzyme that will generate 1.0 μmol of H_2_O_2_ min^−1^ at pH 8 at 37 °C. Each sample was analyzed in quintuplicate. This assay was repeated twice using cells from two rabbits.

### Immunofluorescence staining

Differentiated cells on chamber slides were fixed with 5% formalin (Fisher Diagnostics, Kalamazoo, MI, USA) for 10 min at room temperature, treated with 100 mm glycine for 10 min to remove any autofluorescence, and blocked for 1 h in 5% goat serum in Tris‐buffered saline solution (Abcam, Cambridge, MA, USA). Mouse anti‐HLA DR (LN‐3; Abcam) and anti‐human CD206 (MCA2155; AbD Serotec, Raleigh, NC, USA) antibodies were used to detect cell surface markers of differentiated cells 10. In humans, M1 macrophages express high levels of major histocompatibility complex (MHC). In contrast, the mannose receptor (CD206) is not expressed in M1 macrophages; therefore, it serves as a useful marker for M2 macrophages 16. Cells were incubated with anti‐HLA DR (1 : 50), anti‐CD206 (1 : 100), or mouse Ig G1 isotype control (Abcam) for 2 h at room temperature, and then incubated with Alexa Fluor 488 goat anti‐mouse IgG (H + L) (Life Technologies) or Alexa Fluor 568 goat anti‐mouse IgG (H + L) (Life Technologies) for 1 h at room temperature at a final dilution of 1 : 500. The slides were counterstained and mounted with ProLong Gold antifade regent with DAPI (Life Technologies). The fluorescent images were obtained using an AxioPlan 2 fluorescent microscope (Carl Zeiss Microscopy, Thornmwood, NY, USA) and axiovision microscopy software version 4 (Carl Zeiss Microscopy).

### Fluorescent beads adsorption assay

Fluorescent latex beads (FluoSpheres Sulfate Microspheres, 4 μm diameter, red fluorescent; Life Technologies) were used to determine adsorption and possible uptake of beads. Before use, the beads were washed two times in sterile cold PBS by centrifugation and resuspended in PBS. For opsonization, 10 μL per well of packed beads was then suspended in rabbit serum (MP Biomedicals, Solon, OH, USA) and incubated at 37 °C for 20 min. After washing twice with PBS, the beads were resuspended in 50 μL per well of PBS and added to differentiated cells on a chamber slide or 24‐well plate. After incubation for 90 min at 37 °C, cells were washed two times with ice‐cold PBS to remove free beads. For fluorescent microscopic observation, cells were fixed with 5% formalin and counterstained with trypan blue for 5 min. For quantification of beads entrapped within cells, fluorescence intensities were measured in a LS 55 Fluorescence Spectrometer (PerkinElmer, Waltham, MA, USA). The excitation and emission wavelengths were set at 580 and 605 nm, respectively. Activities were indicated as the mean of five independent wells. All experiments were biologically repeated using blood from two different rabbits.

### Statistical analysis

Data were expressed as means ± SD. The significance of difference was evaluated with the Student's *t*‐test. Significant differences were set at *P* < 0.01.

## Results

### Cytokine mRNA quantification

**Figure 2:**
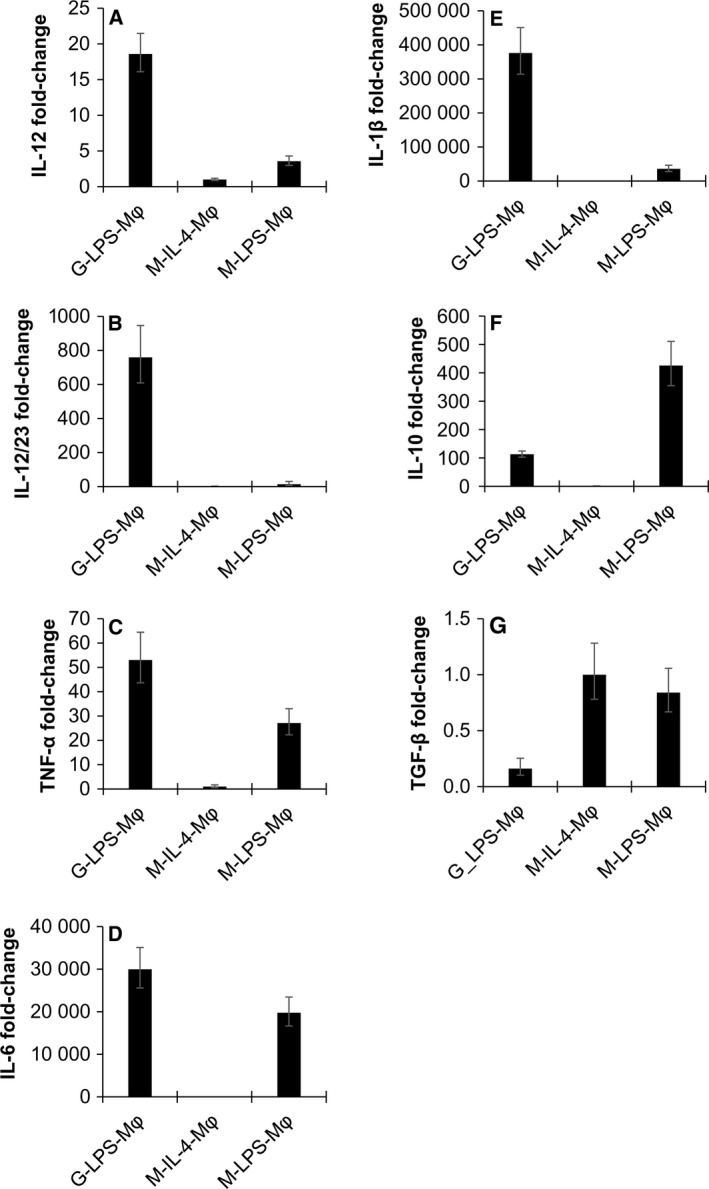
Relative expression of genes encoding marker cytokines for M1 and M2 macrophages. Gene expression profiles of G‐LPS‐MΦ, M‐IL‐4‐MΦ, and M‐LPS‐MΦ were compared using RT‐qPCR. The genes encoding IL‐12 (A), IL‐12/23 (B), TNF‐α (C), IL‐6 (D), and IL‐1 (E) were used as M1 markers. IL‐10 (F) and TGF‐β (G) were used as M2 markers. Blood from two rabbits was evaluated; each sample was analyzed in triplicate.

To characterize polarized cells, mRNA levels of marker genes were evaluated by RT‐qPCR. The mRNA levels of IL‐12 p35, IL‐12/23 p40, IL‐1β, IL‐6, and tumor necrosis factor (TNF)‐α genes were analyzed as M1 phenotypic markers, and IL‐10 and transforming growth factor (TGF)‐β were analyzed as M2 markers. Compared to M‐IL‐4‐ and M‐LPS‐MΦ, G‐LPS‐MΦ samples had higher (*P* < 0.01) mRNA levels for IL‐12 p35, IL‐12/23 p40, TNF‐α, IL‐6, and IL‐1β genes (Fig. 2A–E). Moreover, we observed a high expression of IL‐10 in M‐LPS‐MΦ (Fig. 2F) and TGF‐β in M‐IL‐4‐ and M‐LPS‐MΦ (Fig. 2G).

### NOS activity and arginase activity of differentiated cells

**Figure 3:**
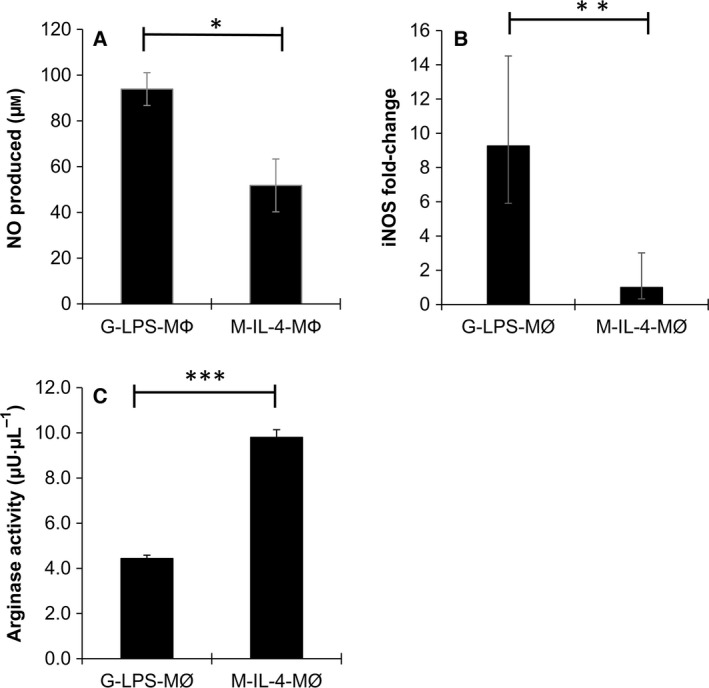
Nitric oxide synthase (NOS) and arginase activities of cell lysates from G‐LPS‐MΦ and M‐IL‐4‐MΦ. The NOS activity of differentiated cells was represented by the accumulation of nitric oxide generated from L‐arginine for 6 h. Five independent wells were analyzed at one time. This assay was repeated two times (A). Relative mRNA expression of the genes encoding inducible nitric oxide synthase (iNOS). Samples from two rabbits were evaluated; each sample was analyzed in triplicate (B). The arginase activity of differentiated cells was represented as the amount of H_2_O_2_ generated by arginase. One unit of arginase is the amount of enzyme that will generate 1.0 μmol of H_2_O_2_ min^−1^ at pH 8 at 37 °C (C). Five independent wells were analyzed at one time. This assay was repeated two times. Statistical analysis was performed using the Student's *t*‐test. *P ≤ 0.01; **P ≤ 0.001; ***P ≤ 0.00001.

Compared to M‐IL‐4‐MΦ, cell lysates from G‐LPS‐MΦ generated significantly larger amounts of NO (Fig. 3A). Moreover, RT‐qPCR revealed significantly higher expression of iNOS mRNA in G‐LPS‐MΦ than in M‐IL‐4‐MΦ (Fig. 3B). In contrast, cell lysates from M‐IL‐4‐MΦ had significantly higher arginase activity than those from G‐LPS‐MΦ (Fig. 3C).

### Surface receptor expression and phagocytic activity of differentiated cells

**Figure 4:**
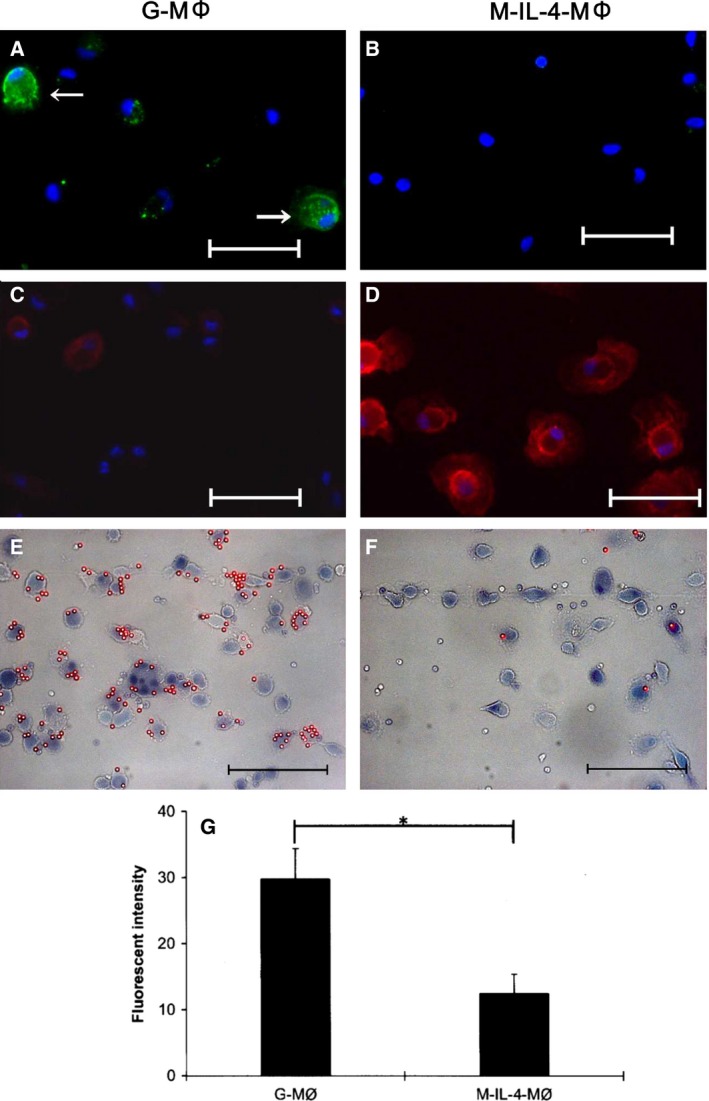
Surface receptor expression and phagocytic activity of differentiated cells. G‐LPS‐MΦs (A, C) and M‐IL‐4‐MΦs (B, D) were fixed with 5% formalin, then blocked and stained with anti‐MHC class II (A, B) or anti‐CD206 (C, D) antibodies. Merged images of fluorescence and counterstaining with DAPI show the expression of MHC class II (A; white arrows) on G‐LPS‐MΦs and CD206 (D) on M‐IL‐4‐MΦs. Uptake of fluorescent latex beads in G‐LPS‐MΦs (E) and M‐IL‐4‐MΦs (F). The cells were incubated with fluorescently labeled opsonized latex beads for 90 min. After washing with phosphate buffer, cells were stained with trypan blue. The fluorescent image is overlaid on phase contrast image of cells. Bars represent 50 μm. The total fluorescence collectively derived from the internalized beads and cell surface‐associated particles was quantified by measuring the fluorescent intensity using a fluorescence spectrometer (G). This assay was performed in quintuplicate and repeated two times. Statistical analysis was performed using the Student's *t*‐test. *P ≤ 0.00001.

For further characterization, G‐LPS‐MΦ and M‐IL‐4‐MΦ were stained with anti‐human MHC class II (green fluorescence) and anti‐CD206 (red fluorescence) antibodies. Some G‐LPS‐MΦ—but not M‐IL‐4‐MΦ—cells stained positively with anti‐human HLA DR antibody (Fig. 4A,B). On the contrary, all M‐IL‐4‐MΦ cells had stronger anti‐CD206‐staining than did G‐LPS‐MΦ cells (Fig. 4C,D).

For uptake of opsonized latex beads, G‐LPS‐MΦ cells were associated with large numbers of latex beads (Fig. 4E), whereas M‐IL‐4‐MΦ cells had substantially fewer bead associations (Fig. 4F). The fluorescent intensity of beads associated with G‐LPS‐MΦ cells was approximately threefold greater (*P* < 0.00001) than the intensity associated with M‐IL‐4‐MΦ cells (Fig. 4G).

## Discussion

The plasticity of macrophages is well‐recognized and has been demonstrated to be dependent on microenvironments, such as cytokines that are produced by a variety of cells. Among these cytokines, GM‐CSF and M‐CSF are known to be crucial not only for survival and proliferation of macrophages but also for determining the fate of macrophage through a development process 17, 18. Human GM‐CSF drives the generation of macrophages that produce proinflammatory cytokines in response to LPS and display high antigen‐presenting and tumoricidal capacity (M1 macrophages) 19. Alternatively, M‐CSF, also known as CSF‐1, has been classified as a M2 stimulus. M‐CSF binding leads to receptor dimerization, and autophosphorylation 20. This cascade induces an anti‐inflammatory phenotype that is involved in tissue repair 17. The ability of human GM‐CSF and M‐CSF to initiate macrophage polarization has been demonstrated in both humans and mice. However, little is known about macrophage polarization using human GM‐CSF and M‐CSF in other model animals. Here, we used human GM‐CSF and M‐CSF to polarize rabbit macrophages from peripheral blood monocytes, because there are no commercially available rabbit recombinant proteins for polarization of rabbit macrophages. When applied to murine macrophages, recombinant human M‐CSF induces the M2 phenotype, but human GM‐CSF does not induce the M1 phenotype in mice 21. Human M‐CSF and GM‐CSF share 83% and 69% amino acid identity with corresponding regions of rabbit M‐CSF and GM‐CSF, and 81% and 55% with mouse, respectively (data from National Center for Biotechnology Information; http://www.ncbi.nlm.nih.gov/). We demonstrated that similarities in the amino acid sequences of growth factors observed among humans and rabbits enabled us to polarize rabbit macrophages to specific subtypes as described below.

Initially, we evaluated effects of recombinant human GM‐CSF‐ and M‐CSF on rabbit macrophages by measuring cytokine mRNA expression patterns. Compared to M‐CSF‐induced macrophages, higher mRNA levels were found for proinflammatory cytokines such as IL‐12 p35, IL‐12/23 p40, TNF‐α, IL‐6, and IL‐1β genes in LPS‐stimulating G‐MΦ but not in M‐MΦ. By contrast, high expression of IL‐10 (immunoregulator) and TGF‐β (tissue remodeler) were observed in rabbit M‐MΦ. Little gene expression of IL‐1β was observed in M‐MΦ. The polarized macrophages phenotypes exhibited by rabbit macrophages were similar to that of human and mouse macrophages based on cytokine profiles and other parameters measured 22. In this study, we maintained having M‐CSF during the stimulation step (Fig. 1). This might compensate for the weaker effects of using human M‐CSF alone to drive the rabbit M2‐polarization.

The M1 and M2 macrophages are also distinguished by the differential expression of diverse molecules such as those associated with the metabolism of arginine 23. In M1 macrophages, iNOS is upregulated, resulting in the catabolism of arginine to NO, the latter plays a key role in the killing of intracellular pathogens 24. Consistent with the findings reported in the literature, rabbit G‐MΦ generated larger amounts of NO than lysates from rabbit M‐MΦ. Different members of the NOS family are encoded by separate genes. The expression of the NOS2 gene that encodes iNOS in G‐LPS‐MΦ was significantly higher than in M‐IL‐4‐MΦ. By contrast, arginase‐1 is induced in rabbit M2 macrophages. This induction results in the production of urea, polyamines, and ornithine that are important for the wound healing actions of this macrophage population 23, 25. Our results suggest that rabbit polarized macrophages displayed similar functional characteristics as those reported for M1 and M2 macrophages derived from other animal species including humans and mice.

In humans, M1 macrophages express high levels of MHC, costimulatory molecules, and FcγR 26. M2 macrophages are characterized by high surface expression of IL‐4R and FcεR, Dectin‐1, CD163, CD206, CD209, and other scavenger receptors. MHC class II and CD206 have been used to show the M1/M2 ratio in rabbit diabetic wounds 9. Our results showed that G‐LPS‐MΦ cells stained positively with anti‐human HLA DR, suggesting that these cells expressed MHC class II. Previous studies demonstrated that expression of MHC class II is upregulated by IFN‐γ stimulation in RAW 264.7, a mouse macrophage cell line 27. Because we stimulated G‐MΦ by combination of LPS and IFN‐γ, further studies using other surface markers are needed to determine the existence of other M1‐type markers on G‐MΦ cells. On the contrary, M‐MΦ cells had stronger anti‐CD206‐staining than did G‐MΦ cells. Therefore, M‐MΦ cells expressed M2 marker molecules on their cell surface. The results on immunostaining of key surface markers on these rabbit macrophage phenotypes are agreeable with the evidence reported in the literature.

M1 macrophage has a higher expression of FcγRs and the ability to bind and uptake opsonized particles 26. The result from the current assay is not possible to distinguish internalized and cell surface‐adsorbed beads although the fluorescent images did show that larger numbers of latex beads were associated with G‐MΦ cells (M1) when comparing with those of M‐MΦ (M2). Technically, one can delineate the source of fluorescent signals by collecting signals resulting only from fluorescently labeled latex beads captured on cell surfaces by flow cytometry. This can be accomplished by incubating cells with fluorescently labeled particles at 4 °C. At this temperature, the attachment of particles to cell surfaces can occur but without subsequent engulfment. By contrast, fluorescence signals from surface‐associated and internalized labeled particles can be obtained by doing the same experiment but done at 37 °C. At 37 °C, both attachment and phagocytosis can take place. Therefore, the resulting difference in the amount of fluorescence between experiments done at these two different temperatures is indicative of signals derived primarily from the internalized particles.

Taken together, our results fully support the notion that human GM‐CSF‐primed rabbit macrophages derived from peripheral blood monocytes can polarize toward a M1 phenotype by IFN‐γ/LPS stimulation. Our data also demonstrated that human M‐CSF‐primed rabbit macrophages had a M2 phenotype. The study described focused on *in vitro* characterization of rabbit macrophages of different phenotypes, which could expand the scope of *in vitro* methods for polarization of human and mouse macrophages. The study also provides additional experimental data to further develop experimental guidelines for macrophage activation and classification 28.

In view of differences could exist between tissue macrophages and *in vitro*‐derived macrophages 29; therefore, it would be interesting to extend the current study to determine the role of M1/M2 ratio during the course of inflammation, infections, and wound healing using rabbit as human disease models. In this regard, there is a study using the rabbit coronary artery disease model illustrating that M1/M2 macrophage ratios could influence disease outcomes 30.

## Author contributions

KY and KPL conceived and designed the experiments. KY performed the experiments and analyzed the data. KY and KPL wrote the manuscript.
